# Optimization of an *in vitro* bioassay to monitor growth and formation of myotubes in real time

**DOI:** 10.1042/BSR20160036

**Published:** 2016-05-06

**Authors:** Sylvia M. Murphy, Maeve Kiely, Philip M. Jakeman, Patrick A. Kiely, Brian P. Carson

**Affiliations:** * Food for Health Ireland, University of Limerick, Limerick, Ireland; † Department of Life Sciences, Materials and Surface Sciences Institute and Stokes Institute, University of Limerick, Limerick, Ireland; ‡ 4i Centre for Interventions in Infection, Inflammation and Immunity, Graduate Entry Medical School, University of Limerick, Limerick, Ireland; § Physical Education and Sport Sciences, University of Limerick, Limerick, Ireland; ∥ Health Research Institute, University of Limerick, Limerick, Ireland

**Keywords:** C2C12, hypertrophy, MPB, MPS, RTCA, skeletal muscle

## Abstract

The importance of growth and maintenance of skeletal muscle is vital for long term health and quality of life. Appropriate nutrition with specific bioactivities relevant to the functionalities of tissues such as skeletal muscle, can assist in maintaining and promoting adaptive responses to biological and environmental stresses which prevent muscle atrophy and promote hypertrophy. The aim of this investigation was to develop a novel *in vitro* cell-based electric impedance assay to study myoblast to myotube formation on the real time cell analysis (RTCA) platform (xCELLigence™, ACEA) and to validate the system by testing myotube responses to hypertrophic stimuli. C2C12 myoblasts were proliferated until 70% confluent in Dulbecco's Modified Eagles Medium (DMEM) (10% FBS) and subsequently differentiated to myotubes over 8 days in DMEM [2% horse serum (HS)]. Changes in cell behaviour and adhesion properties were monitored by measuring impedance via interdigitated microelectrodes in the base of E-16 cell culture dishes. To establish the suitability of this assay to monitor nutrient regulation of muscle hypertrophy, leucine, a known potent regulator of MPS was then supplemented to the fully formed myotubes in physiologically relevant conditions–0.20 mM, 0.40 mM, 0.6 mM, 0.8 mM and above 1.0 mM, 1.5 mM, 2.0 mM and impedance subsequently monitored. Parallel experiments highlighting alterations in myotube thickness, muscle protein synthesis (MPS) (mammalian target of rapamycin; mTOR) and differentiation (myogenin) were conducted to support RTCA bioassay findings. This *in vitro* bioassay can be used to monitor skeletal muscle behaviour and identify nutrient compounds with bioactivities promoting skeletal muscle hypertrophy, reducing muscle atrophy and thus inform the development of novel nutrient formulations for the maintenance of skeletal muscle.

## Abstract (precis)

In the present paper we have developed, described and validated an *in vitro* bioassay to monitor skeletal muscle proliferation and differentiation. We have also demonstrated the use of this assay to evaluate factors which may affect muscle protein balance.

## INTRODUCTION

Skeletal muscle is the largest organ in the human body comprising of approximately 50% of our total body mass [1]. The function of the skeletal muscle is to maintain the skeleton, moving, breathing and thermoregulation and is also an important determinant in over-all body health [2]. Maintaining healthy muscle mass is a critical factor in metabolic health, bone strength, body weight control and resilience to stress and disease. Muscle mass is regulated by a balance of muscle protein synthesis (MPS) and muscle protein breakdown (MPB), the rate of which can be altered through nutrition, age, exercise and sexual dimorphism [3]. Defects in skeletal muscle growth and metabolism can lead to diseases such as diabetes, obesity, heart disease and rheumatoid arthritis [4–6]. Healthy muscle mass promotes overall general good health, therefore preventing many pathologic conditions and chronic diseases [2].

C2C12 cells are a primary murine myoblast cell line, a subclone of C2 myoblasts. These cells are mononucleated, fusiform structures which progressively fuse to form plurinucleate syncytia that further differentiate in culture to acquire the morpho-functional features of the muscle cells [7–9]. Differentiation of skeletal muscle from myoblasts to myotubes *in vitro* is an important tool to facilitate investigations of the mechanisms which regulate muscle mass development and maintenance. The C2C12 cell line is a well-established mouse myoblast cell line used widely as an *in vitro* model of skeletal muscle [9–11]. Manipulation of cell culture conditions triggers fusion of the mononucleated C2C12 myoblasts to form multi-nucleated myotube muscle fibres. The relative thickness of these myotubes in response to treatment with either atrophic or hypertrophic agents can be used as an indicator of MPB or MPS [12,13]. However, the end point nature of many traditional cell-based assays, as well as the challenges associated with measuring myotube thickness e.g. difficulty in measurement of very small atrophic or hypertrophic changes, have hindered our ability to fully utilize this cell line as a screening tool for compounds that can have an effect on muscle mass.

The real time cell analysis (RTCA) xCELLigence™ system has previously been investigated as a potential tool to monitor cell behaviour in real time [13–18]. The xCELLigence™ system allows highly sensitive, label free, non-invasive monitoring of cell proliferation and cell behaviour in real time using micro electric cell sensor arrays integrated into the bottom of tissue culture plates [14,19]. Electrical impedance through the sensor electrodes is monitored and changes in impedance indicate changes in adherence and growth of cells. Change in impedance is recorded as a cell index (CI) value which is a relative, dimensionless value representative of the change in impedance divided by the background value. RTCA is highly accurate when compared with end point assays and has the added advantage of regular monitoring of cellular response to compounds over the entire experiment without any interruption [20].

The aim of the present study was to optimize and validate the use of the xCELLigence™ system to monitor myotube formation from undifferentiated myoblast C2C12 cells. Here, we have developed a complete protocol for optimal differentiation of C2C12 cells from myoblasts to myotube muscle fibres using the xCELLigence™ system and validated this against traditional proteomic and microscopy methods. We describe the optimum conditions to ensure maximum formation of healthy myotubes. We have used proteomics throughout to track markers of the full differentiation process. In order to validate the application and sensitivity of this system further, we also describe the dose-dependent effect of a known hypertrophic agent, leucine, on myotubes as monitored on the xCELLigence™ system. We are confident that this method will facilitate future investigation of the atrophic or hypertrophic effects of compounds on skeletal muscle and will be used as a useful tool to inform and expand our current knowledge of the mechanisms regulating MPS and MPB.

## MATERIALS AND METHODS

### Cell culture

C2C12 cells, a sub clone of C2 myoblasts, were obtained from ATCC® CRL1772, Manassas, VA (Lot number 60339292). Cells were cultured in a 10% complete media (CM), Dulbecco's Modified Eagles Medium (DMEM) (D6429, Sigma–Aldrich) supplemented with 10% (v/v) FBS (F7524, Sigma–Aldrich), 1% (v/v) penicillin/streptomycin (P0781, Sigma–Aldrich) and 1% L-glutamine (G7513, Sigma–Aldrich). Undifferentiated myoblasts were maintained at 37°C in a 5% CO_2_ humidified atmosphere. To induce myoblast fusion, cells were allowed to reach 70% confluence before being switched to a differentiation media (DM),–DMEM (D6429, Sigma–Aldrich) supplemented with 2% (v/v) horse serum (HS). Myotube progression was monitored over the next 8 days until full myotube formation was achieved. Media was changed every 24 h, until the day of testing whereby compounds of interest were added.

### Preparation of cellular protein lysates

Cellular protein extracts were prepared by placing cells on ice, removing media and washing three times in ice cold phosphate buffered saline. Cells were scraped into ice cold lysis buffer (10 mM Tris/HCl pH 7.4, 150 mM NaCl, NaF, 1% NP40) plus the tyrosine phosphatase inhibitor Na_3_VO_4_ (1 mM), protease inhibitors phenylmethanesulfonyl fluoride (PMSF) (1 mM), pepstatin (1 μM) and aprotinin (1.5 μg/ml). Lysates were incubated on ice for 20 min before centrifugation at 130 ***g*** for 15 min at 4°C to remove nuclei and cellular debris. Lysates were analysed for protein concentration using the Bradford assay and boiled in sample buffer for SDS/PAGE (sodium dodecyl polyacrylamide gel electrophoresis).

### Western Blot analysis

Equal amounts of total protein were run on a 12% running and a 4% stacking SDS polyacrylamide gel. Proteins captured in the gel were transferred to a nitrocellulose membrane. The membrane was blocked for 1 h in 5% skimmed milk (w/v) in TBS-T containing 0.5% Tween-20. Membranes were incubated overnight at 4°C in a primary antibody. Primary antibody dilutions were anti-myogenin 1:1000 TBS-T/5% skimmed milk (Santa Cruz (F5D: sc12732)), anti-mTOR 1:1000 TBS-T/5% skimmed milk (Cell Signalling (7C10)). Membranes were then washed for 5 min in 0.05% T-BST, repeated three times, and incubated with an appropriate secondary antibody (IRDye® 680LT and 800CW-Infrared Dye coupled anti-rabbit or anti-mouse, I-COR Biosciences). Antibody reactive bands were detected with the Odyssey® infrared imaging system (LI-COR Biosciences). Band intensities were quantified using Odyssey CLx Western Blot Developer System.

### Cell index measurement

Label free, non-invasive, electric impedance measurements were taken using the xCELLigence™ RTCA Instrument from ACEA. Using a proprietary microelectronic E-16 View gold plated base sensor plate developed by ACEA Biosciences, an automated reading of cell status is taken in real time throughout the myoblast to myotube formation cycle. C2C12 myoblasts were seeded at a density of 0.5 × 10^4^. CI recordings were taken every 15 min during the myoblast proliferation and myotube formation.

### Hypertrophic analysis

Myotubes were formed as described above. On the day of experimentation, myotubes were amino acid and serum starved for 4 h prior to the addition of a range of concentrations of leucine diluted in amino acid free media. Amino acid free DMEM media (D9807-10) was purchased from Life Technologies supplemented with 1% (v/v) penicillin/streptomycin (P0781, Sigma–Aldrich), 1% sodium pyruvate (Thermo Fisher), 1% L-glutamine (G7513, Sigma–Aldrich) and 6 mM D-glucose (G7201 Sigma). Leucine (L8912 Sigma) was purchased from Sigma–Aldrich. CI was recorded every 15 min throughout the duration of the experiment.

### Phase microscopy–hypertrophy/atrophy analysis

Images of myogenic differentiation were taken using Olympus cellSens Dimension 1.12. Copyright© 2009–2014 OLYMPUS CORPORATION Core Version XV 3.11 (Build 12849) Package number 5900 (cellSens Dimension). Labelling and detection probes Nucblue live stain (R37605) and Actin green 488 (R37110) was purchased from Life Technologies. Images were saved as JPEG images and analysed using ImageJ to measure changes in myotube thickness.

## RESULTS

### Determination of the optimal cell number and differentiation time of C2C12 cells on the RTCA xCELLigence™ System

**Figure 1:**
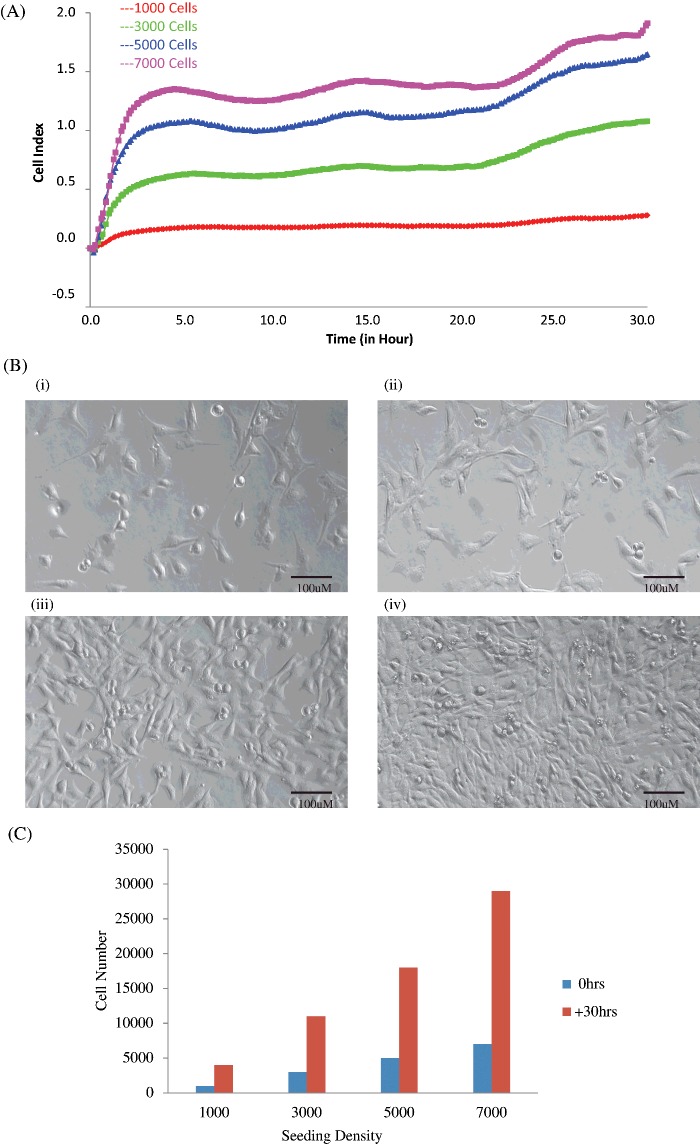
Titration experiment to determine the optimal cell culture number and differentiation time of C2C12 cells C2C12 cells were seeded at a range of titrations–1000, 3000, 5000 and 7000 in duplicate on E-16 VIEW plates that were monitored every 10 min for 30 h. (**A**) Representative graph comparing the growth curve (0–30 h) for 1000 (red), 3000 (green), 5000 (blue) and 7000 (purple) cells. (**B**) Images representing E-16 VIEW plates that were observed using phase contrast for–(i) 1000, (ii) 3000, (iii) 5000 and (iv) 7000 cells at 30 h. (**C**) Cells were trypsinized at 30 h and counted for each seeding density demonstrating cell proliferation from 0 to 30 h. Data are presented as mean ± S.D.

To determine the optimum cell number to achieve the best time to begin myotube differentiation on this platform (70% confluency in high serum media (7)), a titration experiment was carried out (Figure 1A) to monitor myoblast behaviour. Myoblasts were seeded at densities of 1000 cells per well up to 20000 cells per well (all data not shown). Significantly different behaviour was observed when a variety of seeding densities were used on the xCELLigence™ platform (Figure 1A). Myoblasts were cultured using E-16 VIEW plates in a 10% FBS media, and monitored until they reached 70% confluence. These plates have rows of microelectrode sensors removed within the centre of the base of the plate to facilitate clearer imaging of cell performance while still enabling impedance measurement from the xCELLigence™ platform. 70% confluency was achieved after 30 h when cells were seeded at 5000 cells per well (Figure 1B (iii)). Using traditional growth assays, we illustrate that cells are proliferating during this time (Figure 1C).

**Figure 2:**
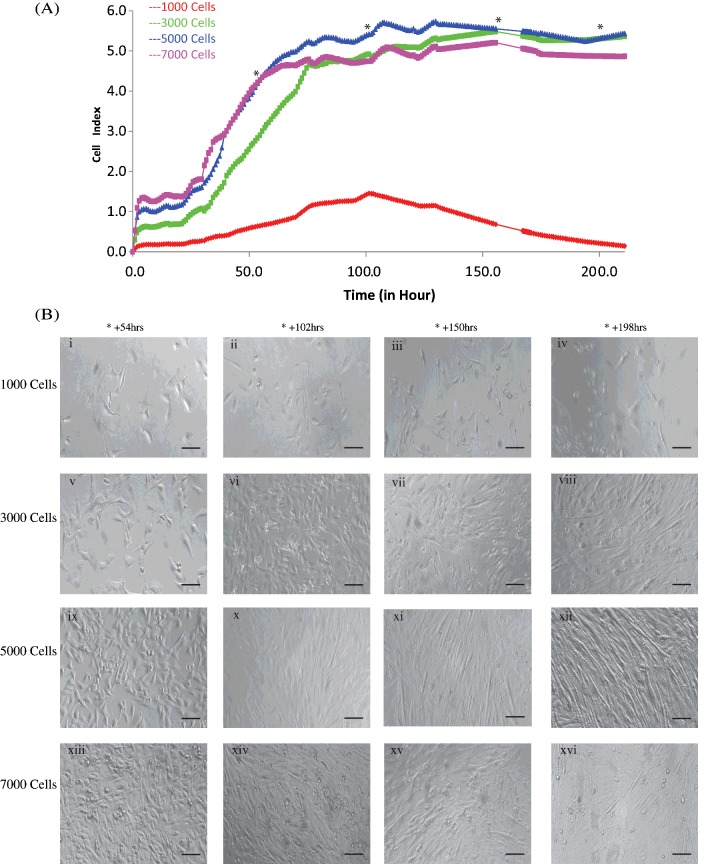
Formation of myotubes over 220 h at different seeding densities C2C12 cells were seeded at a range of densities–1000 (red), 3000 (green), 5000 (blue), 7000 (pink) cells in duplicate in E-16 VIEW plates and monitored every 10 min over 220 h. (**A**) CI of myoblast to myotube formation over 220 h. (**B**) Images representing cells seeded at 1000, 3000, 5000, 7000 all of which were differentiated at 30 h and imaged on Day 1 (+54 h), Day 3 (+108 h), Day 5 (+150 h), Day 7 (+208 h). Scale bars are 100 μM.

After 30 h of proliferation, the 10% FBS media was replaced with a differentiation media containing 2% HS media (see Materials and Methods). This facilitates the fusion of mononucleated myoblasts to form multi-nucleated, elongated, post mitotic myotubes. An overview of the cell performance to complete progression of differentiated myotubes on the RTCA platform after changing to differentiation media (Days 0–8) is shown in Figure 2(A). This progression was monitored over the duration of the assay in parallel using traditional phase contrast imaging at key time points (Figure 2B). The xCELLigence™ graph indicated that 5000 cells per well out-performed all other cell densities, showing a higher CI and provided a more suitable ratio for myoblast fusion and optimal conditions for myotube formation. Phase contrast images of the different cells demonstrate progression of myoblasts to myotubes using different seeding densities (1000, 3000, 5000 and 7000) taken on Days 1 (+54 h), 3 (+108 h), 5 (+150 h) and 7 (+208 h) of the assay. This supports the RTCA data and illustrates that a seeding density of 5000 cells/well is optimal for myotube formation over 8 days. Plating at higher cell densities (≥7000) resulted in cells reaching myoblast cell saturation and confluency at 30 h. These myoblasts were overcrowded and the cells appeared unhealthy resulting in a significant increase in cell death and sub-optimal myotube formation (Figure 2B (xiii–xvi)). Cells seeded at a density below 5000 took a longer period of time to proliferate to 70% and myotube formation is significantly hindered (Figure 2A (red line)). Both high and low densities will ultimately limit the performance and timing of subsequent assays. Our results show that seeding C2C12 skeletal muscle cells at 5000 cells per 0.32 cm^2^ well area enabled proliferation to 70% confluency earlier than either 1000/3000 cells when differentiated at this number and time point, displayed higher viability and improved myotube formation than higher seeding densities (≥7000). This seeding density was adopted as optimal for all further experimentation. This is in agreement with the findings of previous muscle cell culture research [7,13].

### Validation of the xCELLigence™ system as a tool to measure skeletal muscle cell behaviour

**Figure 3:**
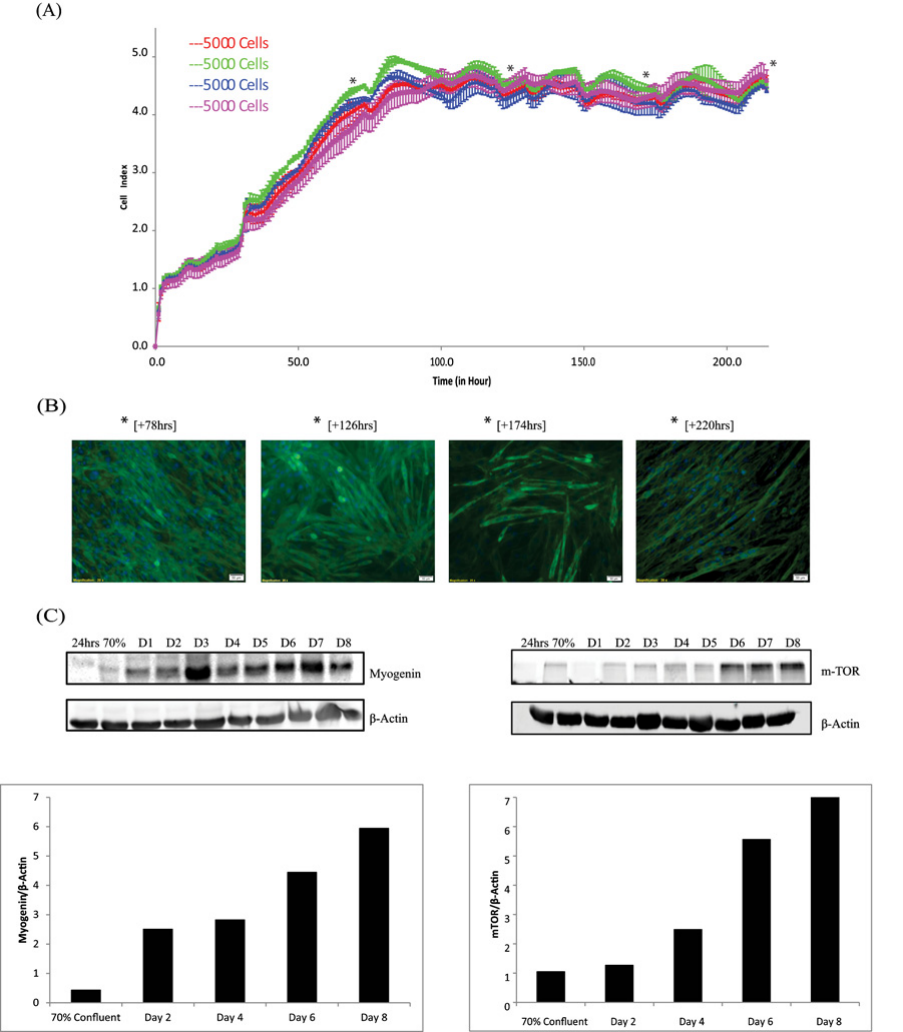
Formation of myotubes and markers of differentiation over 220 h at optimal seeding density (**A**) Represents CI of myotube formation using 5000 cells per well on the xCELLigence™ over 220 h. (**B**) Fluorescent images with actin (green) and nuclear (blue) staining representing cells seeded at 5000 cells per well and imaged on Day 2 (+78 h), Day 4 (+126 h), Day 6 (+174 h) and Day 8 (+220 h). (**C**) Proteomic analysis of markers of myotube differentiation (myogenin, mTOR) over 220 h extracted from lysates taken every 24 h throughout myotube formation.

Having optimized the performance of C2C12 skeletal muscle cells on the xCELLigence™ platform, the next step was to validate the system's measurement of cell behaviour. To monitor the robustness of the myotube formation at 5000 cells/well, we seeded several different wells and examined the growth profile by RTCA. As expected, all wells returned identical graphs with low standard deviations and high levels of reproducibility (Figure 3A). After seeding the cells, we observe an increase in CI in the initial 2–3 h which represents cell adhesion to the plates (Figure 3A). From this point to 30 h we observe an increase in CI due to increased cell number as a result of proliferation (Figure 1C). From 30 to 78 h (Days 1–3), we see no further proliferation or increase in cell number (Figures 2B and 3B), however, we observe an increase in CI due to the initial fusion of cells that occurs during differentiation (Figure 3A). From Days 4 to 8 (to 220 h) we see no further increase in CI (Figure 3A). We use microscopy to illustrate that through the differentiation process, there is myoblast fusion and differentiation to myotubes (Figures 3B). In parallel, we looked at well-known markers of myotube differentiation to validate our RTCA approach (Figure 3C). The mammalian target of rapamycin (mTOR) is a key regulator of protein synthesis and muscle formation and myogenin is an established marker of skeletal muscle differentiation [21–24]. We examined the expression levels of mTOR and myogenin on each day of proliferation and differentiation (Figure 3C) and found an increase in the expression of both pathways that correlated directly with the timing of our myotube formation. Taken together, these data indicate that we can seed C2C12 cells at 5000 cells/well and differentiate over 8 days using the RTCA platform to monitor their differentiation into myotubes that can be validated using known biochemical checkpoints and microscopy.

### Validation of the use of the xCELLigence™ platform to monitor myotube size as illustrated using a known hypertrophic agent

**Figure 4:**
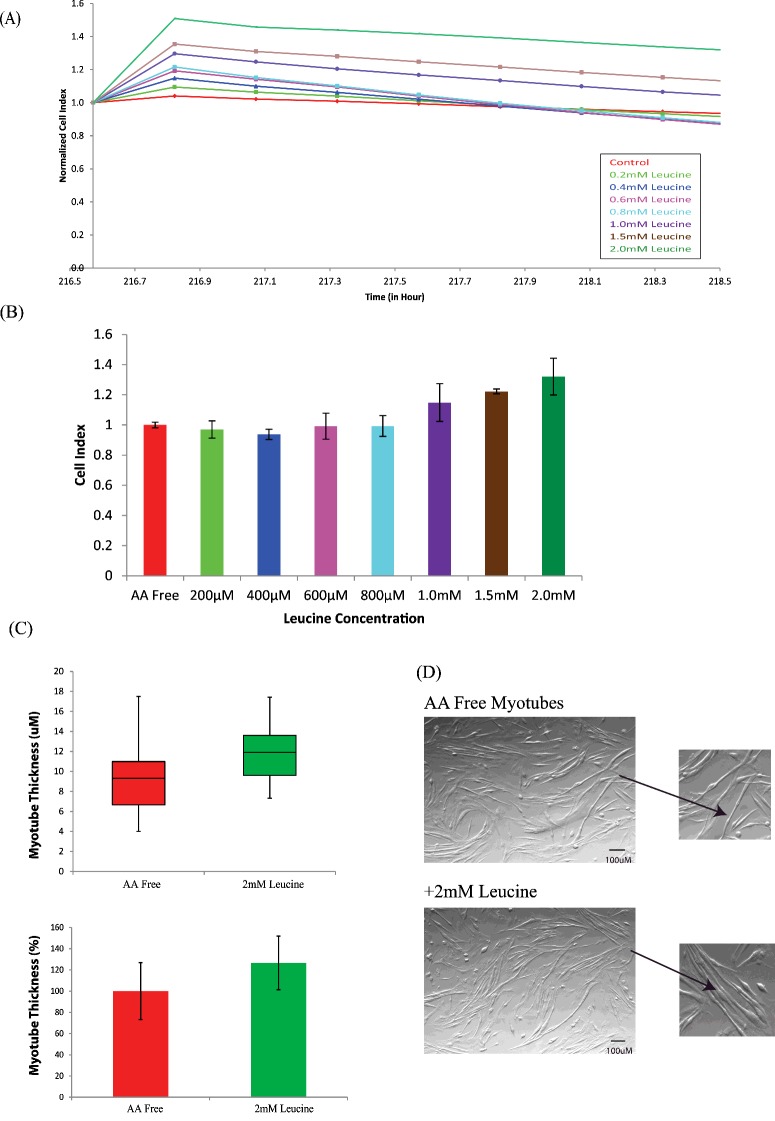
Validation of the sensitivity of myotube performance on the xCELLigence™ system using a known hypertrophic stimuli (**A**) CI was normalized on Day 8 prior to the addition of 200 μM (light green), 400 μM (dark blue), 600 μM (pink), 800 μM (light blue), 1.0 mM (purple), 1.5 mM (brown) and 2.0 mM (dark green) leucine. Dose dependent changes in CI were measured every 15 min post addition of leucine (*n*=4). (**B**) Quantification of CI changes relative to hypertrophic response to different concentrations of leucine. (**C**) Box plot and bar chart comparing myotube thickness as measured by microscopy 4 h post the addition of amino acid free media and 2 h post the addition of 2 mM leucine in absolute and relative thickness respectively. Data are presented as mean ± S.D. (**D**) Phase contrast images taken 4 h post amino acid free media and 2 h post the addition of 2 mM leucine.

To establish the suitability of this assay to monitor nutrient regulation of muscle hypertrophy in physiologically relevant conditions, C2C12 skeletal muscle myotubes were serum and amino acid starved for 4 h. Physiologically relevant concentrations of leucine (200 μM, 400 μM, 600 μM, 800 μM), a known potent regulator of MPS, were then added to amino acid free media as described in Materials and Methods. CI was recorded for the next 12 h (2 h shown in Figure 4A). The data were normalized to a point 4 h post amino acid depravation and just prior to the addition of the hypertrophic agent. The dose-dependent hypertrophic changes were measured post addition of leucine. A dose–response effect was observed with a 32% increase in CI after 2 h in the presence of 2.0 mM leucine (Figure 4B). To validate our approach, images of myotubes post addition of leucine were taken at 4× magnification and myotube thickness was measured using ImageJ [25] and compared with images taken prior to the addition of leucine. We observed a 27% increase in cell thickness after 2 h in the presence of 2.0 mM leucine when compared with control myotubes (Figure 4C). This clearly demonstrates the ability of the xCELLigence™ system to measure myotube size changes in response to hypertrophic stimuli.

## DISCUSSION

The purpose of the present study was to develop a bioassay to monitor growth and formation of muscle myotubes in real time using the xCELLigence™ Real Time Cell Analyser (RTCA) instrument. The primary outcome of the present study is a detailed methodology of the seeding, proliferation and differentiation of C2C12 skeletal muscle cells to fully formed myotubes. We have also validated the xCELLigence™ system for the monitoring of proliferation of myoblasts, and for monitoring changes in fully differentiated myotubes in response to a hypertrophic agent (leucine). This confirms the use of RTCA platforms for medium throughput monitoring of muscle cell behaviour.

C2C12 cells have been used in previous studies to investigate muscle behaviour [10,26], but are difficult to monitor because of extreme heterogeneity [13]. This label free, non-invasive platform allows us to measure cell proliferation and myotube performance in uninterrupted monitoring expressed as a CI value. The essence of the system is the microelectronic cell sensor arrays that are integrated into the bottom of the plate wells. Measuring the electrical impedance on the sensor electrodes indicates the changes in the cells on the electrodes and is recorded. The CI is a relative and dimensionless value since it represents the impedance change divided by the background value. Unlike ‘end-point’ assays, the xCELLigence™ allows the monitoring of the entire experiment in real time without interruption. The RTCA instrument has been developed to study cell activity based on electronic detection of biological assay processes, amalgamating molecular and cell biology with microelectronics. Evaluating the electronic properties of sensor surfaces provide important data about the biological status of the cells or proteins present on the sensors. As changes occur in the biological status of cells or proteins the xCELLigence™ Instrument automatically measures the corresponding development in the electronic properties of the sensors near the cells. These analogue electronic readout signals are then converted to digital signals for processing and analysis [27].

**Figure 5:**
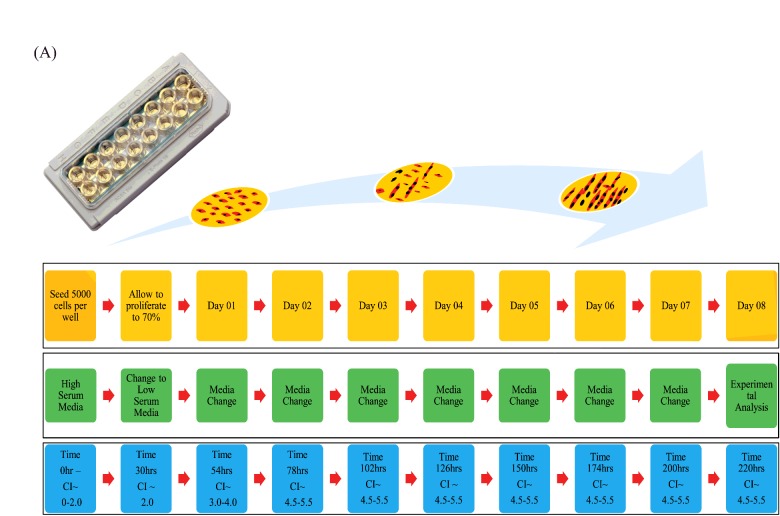
Schematic overview of bioassay Diagram represents a methodological overview of bioassay, highlighting media changes at critical time points and CI guidelines in parallel to each stage of growth from day of cell seeding to full myotube differentiation and hypertrophic analysis on Day 8.

We first established a cell number suitable for the instrument using a titration assay. Five thousand cells were demonstrated as the best performing condition within the E-16 Plate as this seeding density enables timely adhesion of the cells to the plate, progression to 70% confluency within 30 h and increased myotube formation and cell viability. Once myoblasts reached 70% confluence (30 h), growth factors in the media were reduced and differentiation of C2C12 cell myoblasts began. Myoblasts withdraw from the cell cycle and fuse to form post mitotic multinucleated, elongated myotubes. We monitored the myotube progression in parallel using the xCELLigence™ system supported by microscopy, and proteomics using a well-known marker of MPS, mTOR a critical signalling pathway involved in regulating protein synthesis [28–33], and myogenin, a muscle specific transcription factor involved in myogenesis [24]. Western Blot analysis showed an increase in expression of mTOR and myogenin as myotubes were cultured indicating progressive myotube formation and increase in size (Figure 3C). The entire process is illustrated by schematic overview (Figure 5).

Leucine, a known potent regulator of MPS [34–37] was used to validate the sensitivity of the bioassay to myotube size. Different physiologically relevant concentrations of leucine were applied on to the fully formed myotubes after 8 days. Myotubes were monitored every 15 min post application. An increase in CI observed indicated a dose-dependent rise in myotube thickness in response to Leucine. A comparable increase in myotube diameter was confirmed by microscopy in response to leucine using ImageJ software [38].

Great advances have been made in understanding the regulation of skeletal muscle mass both molecularly and physiologically but the scientific community lack robust reproducible assays to monitor muscle formation and performance in the presence of test compounds using miniaturized and medium to high throughput assays. In the present study we illustrate that we can accurately and sensitively trace small changes in myoblast cell morphology behaviour in real time through myogenic differentiation and myotube formation in response to a hypertrophic agent. The practical implications of the present study are critical, suggesting that hypertrophic dietary supplements and recovery compounds for both the aging population and post exercise patrons could potentially be tested on this reproducible, high throughput bioassay.

Here we have described an experimental design and workflow (Figure 5) that allows us to monitor the differentiation and performance of muscle myotubes in real time over 8 days and a post differentiation period in which myotube size/behaviour can be monitored. This method represents a more accurate and in depth account of the C2C12 differentiation process on this real time platform. The application of real-time myogenic analysis could further help investigate changes in muscular mass when subject to hypertrophic/atrophic compounds which would prove salubrious when applied to growth and recovery supplement to promote healthy muscle mass.

## Abbreviations

CI: cell index
DMEM: Dulbecco's Modified Eagles Medium
HS: horse serum
MPB: muscle protein breakdown
MPS: muscle protein synthesis
mTOR: mammalian target of rapamycin
Na_3_VO_4_: sodium orthovanadate
NP40: Nonidet P-40
RTCA: real time cell analysis
